# Extraction of DNA from captive‐sourced feces and molted feathers provides a novel method for conservation management of New Zealand kiwi (*Apteryx* spp.)

**DOI:** 10.1002/ece3.3795

**Published:** 2018-02-17

**Authors:** Ana Ramón‐Laca, Daniel J. White, Jason T. Weir, Hugh A. Robertson

**Affiliations:** ^1^ Landcare Research Auckland New Zealand; ^2^ School of Biological Sciences University of Western Australia Perth WA Australia; ^3^ Department of Biological Sciences University of Toronto Toronto ON Canada; ^4^ Department of Ecology and Evolution University of Toronto Toronto ON Canada; ^5^ Department of Conservation New Zealand Government Wellington New Zealand

**Keywords:** *Apteryx* spp., conservation, feces, low‐template DNA, microsatellites, noninvasive

## Abstract

Although some taxa are increasing in number due to active management and predator control, the overall number of kiwi (*Apteryx* spp.) is declining. Kiwi are cryptic and rare, meaning current monitoring tools, such as call counts, radio telemetry, and surveys using detection dogs are labor‐intensive, yield small datasets, and require substantial resources or provide inaccurate estimates of population sizes. A noninvasive genetic approach could help the conservation effort. We optimized a panel of 23 genetic markers (22 autosomal microsatellite loci and an allosomal marker) to discriminate between all species of kiwi and major lineages within species, while simultaneously determining sex. Markers successfully amplified from both fecal and shed feather DNA samples collected in captivity. We found that DNA extraction was more efficient from shed feathers, but DNA quality was greater with feces, although this was sampling dependent. Our microsatellite panel was able to distinguish between contemporary kiwi populations and lineages and provided PI values in the range of 4.3 × 10^−5^ to 2.0 × 10^−19^, which in some cases were sufficient for individualization and mark–recapture studies. As such, we have tested a wide‐reaching, noninvasive molecular approach that will improve conservation management by providing better parameter estimates associated with population ecology and demographics such as abundance, growth rates, and genetic diversity.

## 1 INTRODUCTION

Since humans arrived in New Zealand around 800 years ago, distributions and densities of kiwi (*Apteryx* spp.) have been in decline, approaching extinction for some species (Ramstad, Colbourne, Robertson, Allendorf, & Daugherty, 2013; Tennyson, Palma, Robertson, Worthy, & Gill, 2003). In addition to clearing habitat and hunting kiwi for food, humans introduced mammalian predators that have taken extensive advantage of their flightlessness and naivety to mammalian predators (Holzapfel, Robertson, & McLennan, 2008; Peat, 1990). Stoats, ferrets, dogs, and cats remain the major threats to kiwi today (McLennan et al., 1996; Robertson, Colbourne, Graham, Miller, & Pierce, 2011). There were only ca. 73,000 kiwi across five recognized species in 2008 according to the 2008−2018 kiwi recovery plan (Holzapfel et al., 2008), which had reduced further to ca. 68,000 in 2015 (Innes, Eppink, & Robertson, 2015). At present, only 24% of all kiwi are under management regimes to prevent predation and population decline (Innes et al., 2015).

Kiwi are nocturnal, flightless, burrowing birds that are now confined to remote areas of New Zealand, making them difficult to study (see (Germano et al., in press; Weir, Haddrath, Robertson, Colbourne, & Baker, 2016) for detailed maps of contemporary kiwi distributions). Currently available monitoring tools for kiwi include call counts, radio telemetry, and surveys using detection dogs (McLennan et al., 1996; Pierce & Westbrooke, 2003; Robertson & de Monchy, 2012; Robertson & Fraser, 2009). While the information gained from these approaches has been invaluable, they also have some limitations. For example, radio telemetry is both labor‐intensive and requires substantial resources, acoustic monitoring is currently restricted to tracking trends in population sizes rather than absolute values, and dog surveys are not effective in low‐density populations (Robertson & Fraser, 2009). Kiwi conservationists, though, need accurate data regarding the size of the current populations of kiwi and their growth rate under different management systems (Innes et al., 2015). Accurate and cost‐effective monitoring techniques are therefore required to enable informed decision‐making at local levels (Germano et al., in press; Holzapfel et al., 2008).

Noninvasive samples have been used in many ecological and demographic avian surveys (e.g., molted feathers (Rodríguez‐Muñoz, del Valle, Bañuelos, & Mirol, 2015), eggshells (Martín‐Gálvez et al., 2011), regurgitations (González‐Varo & Arroyo, 2014; Marrero, Fregel, Cabrera, & Nogales, 2009), and feces (Rösner, Brandl, Segelbacher, Lorenc, & Müller, 2014)). However, although increasing in popularity (Baumgardt et al., 2013; Pérez, Vázquez, Quirós, & Domínguez, 2011; Segelbacher, 2002), deriving population‐level genetic information from noninvasive samples is not yet widespread in birds. While DNA extracted from bird feces has been used in phylogeographic and sex determination studies (Amada, 2012; Baumgardt et al., 2013; Huang, Zhou, Lin, Fang, & Chen, 2012; Idaghdour, Broderick, & Korrida, 2003; Robertson, Minot, & Lambert, 1999), there are fewer reports of using fecal DNA for estimating population parameters (Rösner et al., 2014). This is in contrast to the extensive fecal DNA work done with mammals (Broquet, Ménard, & Petit, 2006; Eggert, Maldonado, & Fleischer, 2005; Ramón‐Laca, Soriano, Gleeson, & Godoy, 2015).

More recently, several studies have used molted feathers as a source of avian nuclear DNA (Alvarez‐Prada & Ruiz‐García, 2015; Huynen, Lambert, McLennan, Rickard, & Robertson, 2003; Rodríguez‐Muñoz et al., 2015; Vázquez et al., 2012). The majority of these studies take advantage of the residual blood cells that remain inside the feather calamus when the growth of the feather is complete, more specifically at the superior umbilicus, meaning they are protected from the environment, UV light, and microorganisms (Horváth, Martínez‐Cruz, Negro, Kalmár, & Godoy, 2005).

Given the invasiveness of traditional capture–mark–recapture sampling methods, and the difficulty of using these sampling methods on a cryptic, nocturnal bird such as a kiwi (Robertson & Fraser, 2009), the use of noninvasive genetics could be a useful source of information for the management and conservation of wild kiwi populations. Noninvasive sampling has an advantage with threatened, elusive, or culturally sensitive species such as kiwi, with the added benefit of reducing stress to birds (Domingo, Marco‐Sanchez, Marco‐Valle, & Pumarola, 1991; Pérez et al., 2011; Segelbacher & Steinbruck, 2001). Furthermore, genetic information can provide demographic information that traditional monitoring methods cannot yield including genetic diversity, parentage, kinship, offspring dispersal, provenance, and sex ratio, without having to disturb or capture individual birds.

**Figure 1:**
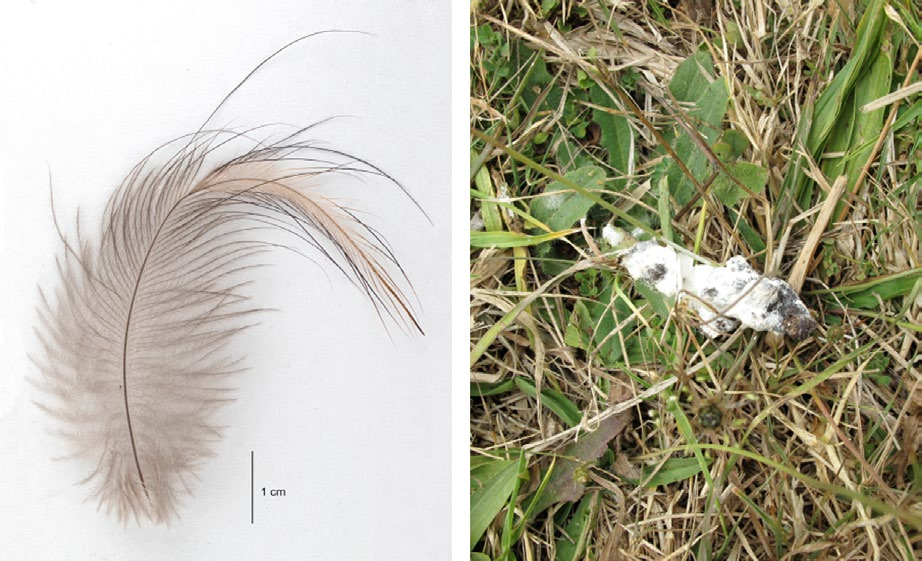
Photo of a barbicel‐lacking feather (left) and kiwi dropping (right)

The aim of this study was the optimization of a large microsatellite marker panel from three preexisting smaller panels (Jensen, Nutt, Seal, Fernandes, & Durrant, 2008; Ramstad et al., 2010; Shepherd & Lambert, 2006), as well as a sex‐determining marker (Dawson, Brekke, Dos Remedios, & Horsbugh, 2015), and its validation as a highly informative, noninvasive genetic tool available for conservation management. To obtain population abundance estimates, as well as relatedness between individuals, it is important that the marker panel has the power to distinguish between individuals. To capture current genetic variation across kiwi, as well as assign individuals to parental populations where necessary, the marker panel must distinguish between the major lineages recently described in Weir et al. (2016). To be noninvasive, the panel must be amplifiable from samples such as feces and shed feathers (see Figure 1), while avoiding frequent genotyping errors, that is, allelic dropout (ADO) and false alleles, found in low‐template DNA samples (Broquet et al., 2006). These errors can lead to inaccurate sex determination or incorrect individual assignments (Baumgardt et al., 2013). Finally, for convenience and to allow for overlapping distributions, the panel must cross‐amplify across all five recognized species—*Apteryx mantelli*,* A. owenii*,* A. rowi*,* A. australis*, and *A. haastii*.

## 2 MATERIALS AND METHODS

Optimization of the marker panel in all recognized kiwi species was followed by performance assessment of different methods of noninvasive sample collection and extraction.

### 2.1 Optimization of microsatellite marker panel

**Figure 2:**
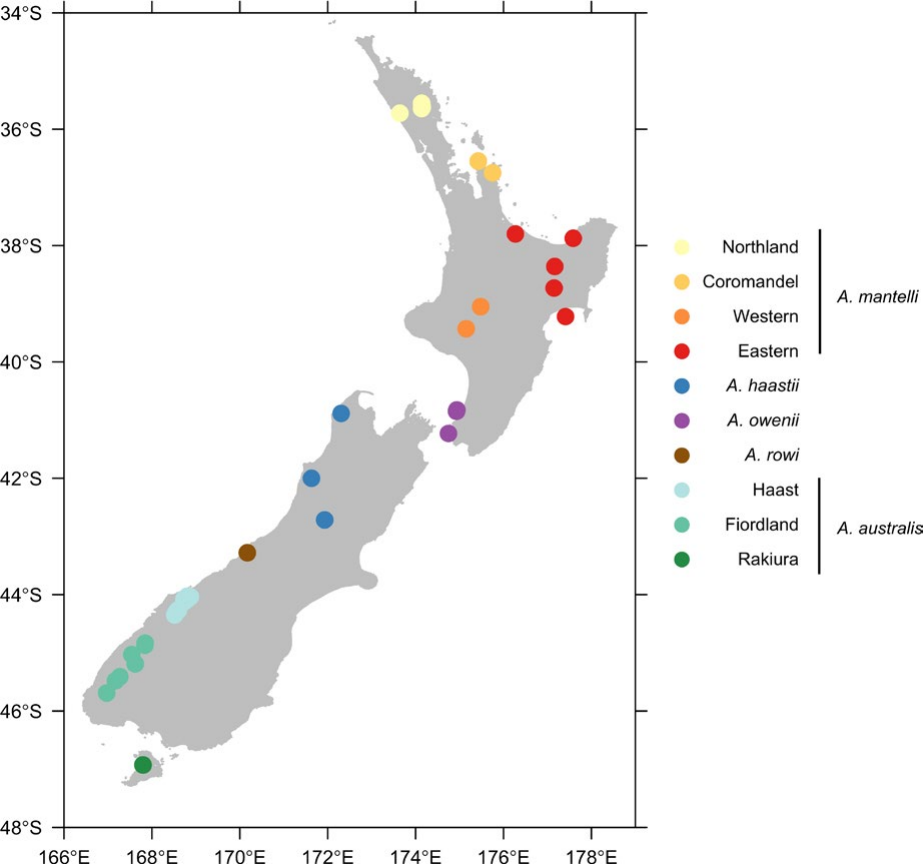
Map showing the distribution of 170 out of 326 kiwi used in this study

A selection of 22 previously described microsatellite loci (Jensen et al., 2008; Ramstad et al., 2010; Shepherd & Lambert, 2006) were tested on 326 DNA samples extracted from five kiwi species—233 *A. mantelli* (15 blood, 11 tissue, 206 feathers, and 1 carcass swab), 3 *A. rowi* (3 blood), 74 *A. australis* (17 blood, 54 feathers, and 3 carcass swabs), 7 *A. owenii* (7 feathers), and 9 *A. haastii* (3 blood and 6 feathers). These samples were collected within natural distributions for each species (apart from *A. owenii* which is now confined to offshore islands), from around New Zealand (Figure 2). Sex was determined by amplification of an EST‐derived microsatellite locus for birds, Z37B (Dawson et al., 2015), which produces short fragments that are different in size for W and Z chromosomes. To assess reliability and accuracy, the Z37B primer set was tested on known‐sex samples from *A. mantelli* (11 females and 23 males), *A. owenii* (4 females and 3 males,), *A. haastii* (2 females and 4 males), and *A. australis* (7 females and 24 males). Finally, sex determination results using the Z37B marker were compared to those obtained using the w5/w7 marker (Huynen et al., 2003) for 37 females and 45 males from *A. mantelli*,* A. australis*, and *A. haastii*, as the w5/w7 marker was designed specifically for *A. australis* and could be superior for sex determination across *Apteryx* spp.

PCR amplifications were performed in a 10 μl final volume, containing 1× Qiagen Type‐it Microsatellite PCR master mix, between 0.1 and 0.45 μmol/L of each primer (Table S1 in Appendix S1), and 1 μl of each DNA extract. Qiagen cycling recommendations were followed (35 cycles, 90 s annealing time, and 57°C annealing temperature). DNA extracts were amplified in three different multiplexed PCRs, from which two (2a and 2b,Table S1 in Appendix S1) were combined for capillary electrophoresis analysis on a genetic analyzer 3500xL (Applied Biosystems), meaning only two electrophoresis runs were required per sample.

### 2.2 Utility of marker panel

To assess the utility of the marker panel for population‐level analyses and individualization, the following metrics were estimated using the genotypes of the above‐mentioned 326 kiwi: average number of alleles per marker, expected and observed heterozygosity, number of population‐specific alleles, and the probability of identity for increasing combinations of loci. To assess how well our marker panel can distinguish between species and major populations and/or provenances, samples representative of the taxonomic groups recently defined by Weir et al. using 6332 SNPs (2016) were analyzed in principal coordinates analyses (PCoA). Analyses were conducted in GenAlEx v6.501 (Peakall & Smouse, 2012).

### 2.3 Noninvasive sample collection and DNA extraction

**Figure 3:**
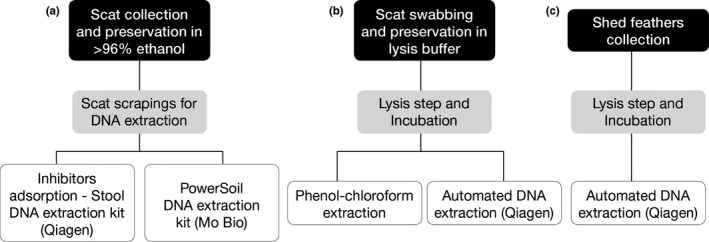
The sampling and DNA extraction protocols trialled. All samples were eluted in 70 μl in the final step. See Appendix S1 for further details

Twenty‐five fresh fecal samples (≤1 day old) that had been protected from rainfall were collected from three captive‐breeding facilities: Westshore Wildlife Reserve, Napier (location 1); Kiwi Encounter, Rainbow Springs, Rotorua (location 2); and Auckland Zoo, Auckland (location 3). A rayon swab was used to collect fecal material (Figure 3b) from the surface of the stool (see instructional video: http://youtu.be/zniEFYLSgOI) (Bosnjak, Stevanov‐Pavlovic, & Vucicevic, 2013; Ramón‐Laca et al., 2014), and immediately on collection, the head of the swab was preserved in ca. 500 μl of lysis buffer (Longmire, Maltbie, & Baker, 1997) to avoid any biotic, hydrolytic, enzymatic, or microbial degradation that could cause ADO. The urine‐associated, white part of the scat was avoided whenever possible, as it was found to inhibit the PCR in a small pilot study (results not shown—see also (Segelbacher & Steinbruck, 2001)); 4.2 μl of DX digestive enzyme (Qiagen) was added to 220 μl of the suspension and incubated overnight at 56°C, followed by an automated extraction in a QIAxtractor instrument using DX reagents (Qiagen). DNA was eluted in 70 μl of elution buffer. Three other sample collection and DNA extraction protocols were also assessed (Figure 3, and see Appendix S1 for more details).

Twenty‐six freshly molted individual body feather samples (≤1 day old) were collected from two of the three captive facilities and were dry‐stored in paper envelopes. In the laboratory, each feather was placed in a 1.5‐ml tube and the calamus soaked in 420 μl DXT buffer and 4.2 μl of digestive enzyme. Again, DNA was extracted from 220 μl of this suspension after an overnight incubation at 56°C (Figure 3c).

### 2.4 Quantification of the DNA

Total DNA quantity of samples was measured using a QuantiFluor‐ST Handheld fluorometer (Promega) and a Picogreen dsDNA dye kit (Quant‐iT, Invitrogen, by Life Technologies). Eight serial dilutions of the λ standard ranging from 25 to 1.5 ng/μl were used to build the standard curve. DNA extracted from molted feathers was not quantified using the fluorometer because the range of DNA extracted fell outside the lower limit of this platform (0.5 ng/μl). Furthermore, the majority of DNA purified from feathers was believed to be of kiwi origin.

DNA extracted from molted feather and fecal samples was then subjected to target DNA quantification, enabling target‐to‐total DNA ratio to be estimated for the fecal samples. A monomorphic microsatellite, KMS16B (Jensen et al., 2008) of 148 base pairs, was used to quantify the target DNA in a quantitative PCR (qPCR) approach. Ten serial dilutions from 5 to 0.002 ng/μl were used as standards for the quantification. All samples and standards were run in triplicate on a Rotor‐Gene 6000 (Corbett Research) with a first holding step of 5 min at 95°C, followed by 45 cycles of 5 s at 95°C, 30 s at 60°C, and 30 s at 72°C and a final melting step from 60 to 90°C to evaluate the specificity of the reaction. PCR mixes consisted of 5 μl of LightCycler 480 SYBR Green I mix (Roche), 0.5 μmol/L each of KMS16B forward (CCCCCCACTAAGTCTG) and reverse (AAGTATTCTTGGTAAACAGG) primers (Jensen et al., 2008), 0.4 μg/μl of bovine serum albumin (BSA), and 1 μl of the DNA template in a 10 μl reaction. Samples that failed to amplify were diluted up to 10‐fold and rerun.

### 2.5 PCR inhibition assessment

To assess the level of inhibition in the fecal DNA extracts, a similar protocol to the target DNA quantification experiment was followed, except a lower BSA concentration (0.15 μg/μl), and 2.5 ng/μl of standard was included in the PCR mix as an inhibition control (1 μl per sample). Samples were run in duplicate, and the 2.5 ng/μl positive control (no DNA or standard added) was run in triplicate. Inhibition occurrence was defined as the average *C*_q_ (quantification cycle) of the replicates for each sample minus the average *C*_q_ of the positive control replicates (Δ*C*_q_). Samples are expected to show negative results in the absence of inhibitors and positive values when PCR inhibitors were copurified in the DNA extraction. Samples that failed to amplify were given a *C*_q_ value of 45 (the total possible number of cycles). Inhibition occurrence tests were not performed for the shed feather samples as no PCR inhibitors were assumed to be copurified in the DNA extraction procedure.

### 2.6 DNA quality assessment

To assess the quality of DNA, DNA extracts from the molted feathers and fecal swabs were subjected to microsatellite profiling (22 loci) and sex determination (EST‐derived microsatellite locus) (Table S1, Appendix S1) in quadruplicate. Multiplexed PCR amplifications were performed as for the panel optimization, but with 2 μl of each DNA extract and 45 cycles in the PCR. All microsatellite fragments from noninvasive and invasive samples were scored and edited using GeneMapper v 5.0 (Applied Biosystems). DNA quality was assessed with the consensus quality index (QI) (Miquel et al., 2006) as described in the study of Ramón‐Laca et al. (2015). Genotyping error rates across replicates were estimated using GIMLET v 1.3.3 (Valière, 2002), in which errors were classed as discrepancies of each replica to its consensus profile, or to the consensus profile of the reference feather sample when available. To validate the application of fecal and molted feather samples to population monitoring, noninvasive genotypes were included in a PCoA analysis with genotypes generated from high‐quality reference samples (blood, tissue, plucked feathers, and carcass swabs) using birds of known origin. Any sample that failed at more than two loci was removed from the analysis.

## 3 RESULTS

### 3.1 Gender determination and microsatellite marker development and optimization

All 22 autosomal markers were amplified in four kiwi species, with amplicons that ranged in size from 74 to 357 bp. Of the 22 loci, 18 were polymorphic in *A. haastii*, 15 in *A. rowi*, 13 in *A. owenii*, and 21 in *A. mantelli*. One marker, Aptowe 28, failed in *A. australis*, meaning 21 autosomal microsatellite markers successfully amplified, 18 of which were found to be polymorphic. Fragment lengths, variability, and allele fixation were mostly consistent with previously published results (Table S1) (Jensen et al., 2008; Ramstad et al., 2010; Shepherd & Lambert, 2006). In contrast to Ramstad et al. (2010), locus Aptowe29 successfully amplified and was found to be polymorphic in *A. mantelli* in this study. Marker panel performance and diversity measures are summarized in Table 1. *A. mantelli* showed greatest genetic diversity (Tables 1 and S3). Average numbers of alleles per marker ranged from two (*A. rowi*,* N* = 3 and *A. owenii*,* N* = 7) to 8.7 (*A. mantelli*,* N* = 233), numbers of private alleles ranged from four in *A. rowi* to 69 in *A. mantelli*, observed heterozygosity ranged from 0.23 in *A. owenii* to 0.495 in *A. mantelli,* and the inbreeding coefficient was highest in *A. australis* (0.306), suggesting some of these samples came from related birds (Table 1), which is expected as 49 of 74 *A. australis* samples were from the small (ca 400 bird) Haast population.

**Table 1:** Marker panel performance and diversity measurements in five species of kiwi

Species	Population	*N*	*N*_A_	*A*	*H*_E_	*H*_O_	*F*_IS_	PI_23_ a	PIsibs_23_ a	PI_18_ a	PIsibs_18_ a
***Apteryx mantelli***		**233**	**8.7**	**69**	**0.585**	**0.495**	**0.138**	**2.0 × 10** ^**−19**^	**8.3 × 10** ^**−8**^	**3.9 × 10** ^**−16**^	**1.8 × 10** ^**−6**^
	Northland	34	5	5	0.5	0.5	−0.006	1.6 × 10^**−**14^	1.6 × 10^**−**6^	2.8 × 10^**−**12^	1.5 × 10^**−**5^
	Coromandel	52	4	1	0.521	0.476	0.063	6.0 × 10^**−**15^	9.5 × 10^**−**7^	1.1 × 10^**−**12^	1.1 × 10^**−**5^
	Western	74	6	10	0.526	0.498	0.047	1.5 × 10^**−**16^	5.5 × 10^**−**7^	4.5 × 10^**−**14^	5.9 × 10^**−**6^
	Eastern	73	7	15	0.542	0.505	0.050	3.6 × 10^**−**17^	3.3 × 10^**−**7^	4.8 × 10^**−**14^	6.4 × 10^**−**6^
***Apteryx haastii***		**9**	**4**	**21**	**0.499**	**0.425**	**0.147**	**1.6 × 10** ^**−14**^	**1.5 × 10** ^**−6**^	**1.6 × 10** ^**−11**^	**3.2 × 10** ^**−5**^
***Apteryx owenii***		**7**	**2**	**6**	**0.207**	**0.23**	**−0.128**	**4.3 × 10** ^**−5**^	**6.7 × 10** ^**−3**^	**5.7 × 10** ^**−5**^	**7.7 × 10** ^**−3**^
***Apteryx rowi***		**3**	**2**	**4**	**0.331**	**0.406**	**−0.226**	**1.6 × 10** ^**−8**^	**2.3 × 10** ^**−4**^	**1.3 × 10** ^**−7**^	**6.5 × 10** ^**−4**^
***Apteryx australis***		**74**	**5.7**	**21**	**0.485**	**0.337**	**0.306**	**1.2 × 10** ^**−14**^	**1.9 × 10** ^**−6**^	**8.8 × 10** ^**−12**^	**3.7 × 10** ^**−5**^
	Haast	49	3	3	0.364	0.306	0.165	1.3 × 10^**−**9^	8.6 × 10^**−**5^	2.1 × 10^**−**7^	9.4 × 10^**−**4^
	Fiordland	13	4	5	0.460	0.4	0.119	1.5 × 10^**−**13^	4.0 × 10^**−**6^	2.9 × 10^**−**10^	1.0 × 10^**−**4^
	Rakiura	12	3	1	0.404	0.397	**−**0.003	2.2 × 10^**−**10^	3.3 × 10^**−**5^	2.3 × 10^**−**8^	3.1 × 10^**−**4^

*N*: number of samples; *N*_A_: average number of alleles per marker; *A*: number of private alleles; *H*_O_: observed heterozygosity; *F*_IS_: inbreeding coefficient; PI_23_: probability of identity for increasing locus combinations at all 23 loci, PI_18_: probability of identity for increasing locus combinations at 18 loci (17 autosomal microsatellite markers and Z37B).

a One autosomal microsatellite (Aptowe28) was excluded from *Apteryx australis,* so values described here for this species are PI_22_, PIsibs_22_, PI_17_, and PIsibs_17_. Bold type reflects values for the five recognized species, normal type for recognized taxa within the species.

All 130 known‐sex samples were correctly assigned using Z37B. Sex determination results using Z37B for *A. mantelli, A. owenii,* and *A. haastii* matched those of the w5/w7 primers, apart from one *A. mantelli* individual (1.4% mismatch), and two alleged *A. australis* males from Haast were sexed using w5/w7 primers that returned a female genotype with Z37B. A summary of Z37B fragment lengths per species is detailed in Table 2.

**Table 2:** Results from the sex determination test using Z37B primers

Common name	Species	Size of the fragment (bp)	
		W	Z
North Island brown	*Apteryx mantelli*	92	96, 98,^a^ 100^b^
Tokoeka	*Apteryx australis*	92	96, 98^c^
Rowi	*Apteryx rowi*	92	96
Little spotted kiwi	*Apteryx owenii*	92	94
Great spotted kiwi	*Apteryx haastii*	92	94

ZZ = male; ZW = female; bp, base pairs.

a The 98 bp allele was only observed in the Western and Eastern lineages.

b The 100 bp allele was only observed in the Western lineage.

c The 98 bp allele was only observed in Fiordland.

### 3.2 Utility of marker panel

**Figure 4:**
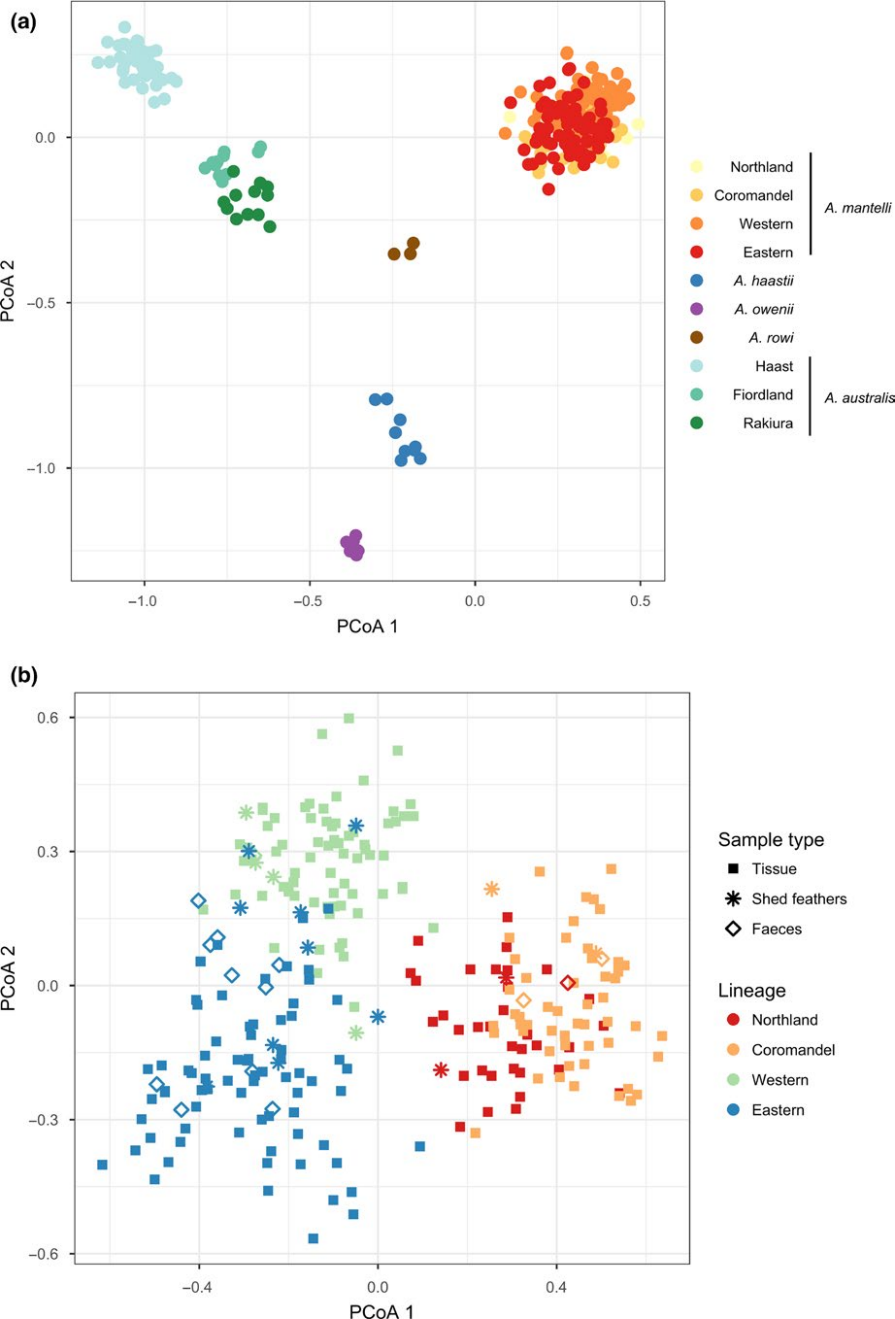
(a) Principal coordinates analysis (PCoA) of unique genotypes for five kiwi species using reference samples and (b) PCoA of unique genotypes for *Apteryx mantelli* using reference and noninvasive samples. The first and second coordinates represent the *x*‐ and *y*‐axes, respectively

The probability of identity for the 23 markers ranged from 4.3 × 10^−5^ for *A. owenii* to 2 × 10^−19^ for *A. mantelli,* and the probability of identity for siblings ranged from 6.7 × 10^−3^ for *A. owenii* to 8.3 × 10^−8^ again for *A. mantelli*. Samples of known origin clustered well into the genetically determined lineages described in Weir et al. (2016) (Figure 4a,b). For *A. australis*, there is good discrimination between Rakiura, Fiordland, and Haast, and moderate discrimination between North and South Fiordland, although the sample size is small for these latter two populations (seven and four, respectively). For *A. mantelli,* there is good discrimination between Northland/Coromandel and East/West and moderate discrimination within each of these clusters.

### 3.3 DNA quantification of noninvasive samples

**Figure 5:**
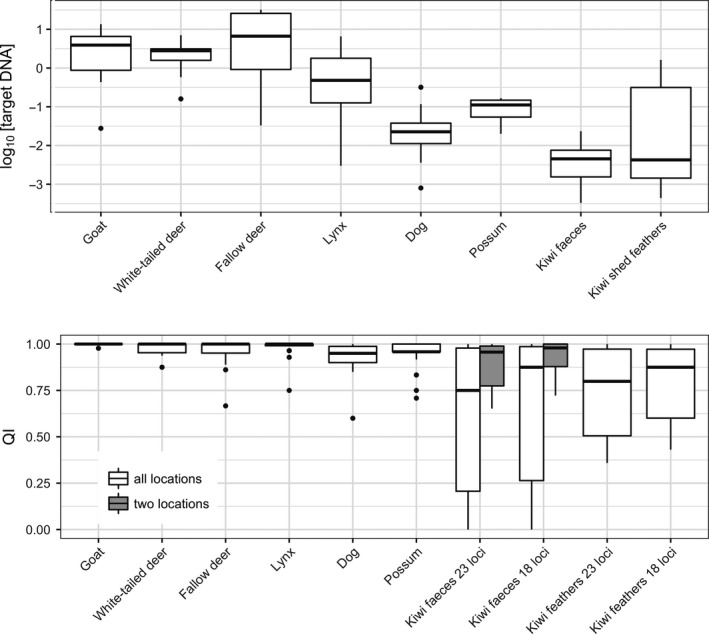
Log_10_‐transformed target DNA concentration (measured in ng/μl) and quality index results for kiwi feces and shed feather samples compared with six mammalian species from (Ramón‐Laca et al., 2015). QI scores range from 0 to 1, where 1 indicates complete agreement between all replicates

Total DNA concentrations for the fecal samples yielded 12.67 (±19.46 *SD*) ng/μl. Average target nuclear DNA concentration for the fecal samples was 9.37 (±13.18 *SD*) pg/μl for all three sampling locations and 6.75 (±7.18 *SD*) pg/μl for the two sampling locations that did not show blatant inhibition (locations 1 and 2—Westshore Wildlife Reserve and Kiwi Encounter, respectively). Target DNA recovery was highly variable among sampling locations as target‐to‐total ratios for locations 1, 2, and 3 were 0.90%, 8.12%, and 0.03%, respectively. Shed feather samples yielded 237 (±421 *SD*) pg/μl of target DNA (Figure 5 and Table S3).

### 3.4 PCR inhibition assessment

PCR inhibitors were found in 6.24% of the samples from the fecal treatment. Δ*C*_q_ = 1.74 (±6.84 *SD*) and was more frequent in one of the three sampling locations (location 3—Auckland Zoo). Δ*C*_q_ is reduced to −0.94 (±0.99 *SD*) if location 3 is excluded, with evidence of inhibitors in only one out of 14 samples (Figure 5 and TableS1).

### 3.5 DNA quality from noninvasive samples

Using 22 microsatellites and the sex determination marker, genotype quality (QI) for the fecal samples was 0.64 (±0.36) for all three locations and 0.88 (±0.13) when the sampling location with the abnormally high PCR inhibition (location 3) is removed. When the five microsatellite loci that had the lowest QI score for the fecal samples were removed from analysis (KMS30, KMS14B, KMS18, KMS7R, and KMS1), QI was 0.69 (±0.4) when all three locations were considered and 0.93 (±0.35) for locations 1 and 2 (Figure 5 and Table S1). We arbitrarily considered a genotype to be successful when its QI was 0.75 or higher. According to this criterion, 52% of the samples were successfully genotyped for 23 loci across all locations, and 71% if only locations 1 and 2 were considered (Table S3); 68% of the samples were successfully genotyped for the 18 best‐performing loci, and 92% if only locations 1 and 2 were considered. QI for the plucked feather samples was 0.74 (±0.23) for all 23 loci and 0.79 (±0.14) for 18 loci; 57.7% of the molted feather samples had QI scores >0.75 for both 18 and 23 loci.

Although relatively high rates of amplification failure and ADO were observed, the false allele rate was negligible (Table 3). Overall, Z37B was the best performing of the 23 markers (Table S3). The PCoA analysis showed that the noninvasive samples clustered well with their respective populations of origin (Figure 4b) with the exception of a few samples from Eastern and Western lineages of *A. mantelli* whose genotypes were incomplete.

**Table 3:** Amplification failure, allelic dropout, and false allele occurrence (%)

Type of sample	Amplification failure	Allelic dropout across loci	Allelic dropout across samples	False alleles across loci	False alleles across samples
Molted feathers	14	21.9	25.1	0.8	0.7
Fecal swabs_3	28	20.5	16.2	1.3	1.9
Fecal swabs_2	6	16.7	12.0	0.8	0.3

Fecal swabs_3: reflects the genotyping error rates of all the fecal swab samples analyzed; Fecal swabs_2: reflects only fecal swab samples from the two best‐performing locations.

## 4 DISCUSSION

A panel of 22 autosomal microsatellite markers and one sex‐determining marker (Z37B) has been tested and cross‐amplified across all five recognized kiwi species from both molted feathers and fecal material. This is the first time that a sexing marker has been optimized for low‐template and poor‐quality DNA in kiwi. Although there was minor disagreement with the sex predicted using w5/w7 (which was designed to help resolve female assignment in *A. australis* (Huynen et al., 2003; Huynen, Millar, & Lambert, 2002)) in one *A. mantelli* case where we predicted the bird to be male and in two *A. australis* cases where in both cases we predicted the bird to be female, nesting behavior records suggest the *A. mantelli* bird to be male (Hugh Robertson pers. comm.), supporting the sex determination using Z37B. While further tests using known‐sex *A. rowi* and Haast *A. australis* samples will be necessary to determine the utility of Z37B for these species, initial results suggest Z37B could be superior to w5/w7 at accurately predicting sex across multiple kiwi taxa.

Maintaining current kiwi genetic diversity, and the integrity of genetic lineages, is a major objective of the 2017–2027 Kiwi Recovery Plan (Germano et al., in press). Although there are five recognized species of extant kiwi, recent work by Weir et al. (2016) used 6332 SNPs to show 11 contemporary genetic lineages and represent the most comprehensive attempt to genetically characterize extant kiwi taxa to date. Our marker panel easily distinguished the five recognized kiwi species and further was also able to discriminate between all four *A. mantelli* provenances (East, West, Northland, and Coromandel) with varying degrees of power and clearly shows three provenances within *A. australis*—Rakiura, Fiordland, and Haast. While North and South Fiordland tended to cluster separately, the delimitation was less clear, which increasing the small number of samples (seven and four, respectively) would likely resolve. In addition to providing base level estimates of genetic diversity in kiwi populations as targets for ongoing conservation efforts, our marker panel may also, therefore, prevent the erosion of current genetic variation. For example, when choosing individuals for translocations and breeding programs, the mixing of pure genetic lineages, which could lead to outbreeding depression, can be avoided.

The marker panel also showed sufficient discriminatory power to separate individuals, especially in species and lineages that show high overall genetic diversity. This means we have the capability to disentangle parentage within kiwi groups and to assess breeding success in translocated and managed populations, particularly for cases where potential parents have already been sampled. For example, breeding success may be a useful indicator for measuring how successful Operation Nest Egg (an ex situ captive rearing program) is at repopulating regions with viable kiwi (Germano et al., in press). Individualization also allows genetic tagging for mark–recapture studies, useful for understanding population densities and size, as well as distribution range (Mowat & Strobeck, 2000; Peakall, Ebert, Cunningham, & Lindenmayer, 2006).

The application of this molecular approach to noninvasive samples raises several challenges. The success of noninvasive genetic approaches is dependent on retrieving sufficient target DNA from samples—in this case the umbilicus clot for feathers (Horváth et al., 2005), and the intestinal epithelial cells which are sloughed from the digestive tract for kiwi feces—while avoiding DNA degradation and copurification of PCR inhibitors (Ramón‐Laca et al., 2015). Four different DNA extraction methods were tested in this study for fecal DNA purification, with only the swabbed feces with automated extraction approach yielding DNA of sufficient quantity and quality for genotyping purposes. Idaghdour et al. (2003) were able to extract ca. 100–120 pg/μl of g DNA from great bustard feces, while we obtained two orders of magnitude less of target nuclear DNA. However, this appeared to be enough to reliably amplify genotypes. While target nuclear DNA concentration and genotype quality achieved here are lower than the values obtained in mammals (see Figure 5 and (Ramón‐Laca et al., 2015)), amplification failure and ADO rates are comparable, and false allele occurrence was lower than in other avian studies (Tables 3 and S2) (Bayard de Volo, Reynolds, Douglas, & Antolin, 2008; Horváth et al., 2005; Johansson, McMahon, & Höglund, 2012; Miño & Lama, 2009; Pérez et al., 2011; Regnaut, Lucas, & Fumagalli, 2006; Rösner et al., 2014; Segelbacher & Steinbruck, 2001; Segelbacher & Storch, 2002).

We found the target DNA yield from molted kiwi feathers to be low compared to other birds (Table S3). Some studies (Bayard de Volo et al., 2008; Johansson et al., 2012; Segelbacher,^2002^; Vili et al., 2013) have found larger feathers (remiges, primaries, and secondaries) to yield more DNA than small feathers (tertiaries, covert, and down), and that large birds generally yield more DNA than smaller birds. In contrast, kiwi have only two types of feathers: bristles (bristles, semibristles, and intermediate forms) without umbilical barbs or barbules, found around the beak and on the forehead (Cunningham, Alley, & Castro, 2011), and barbicel‐lacking, “hair‐like” feathers with long and loose barbs (Figure 1) (Harwood, 2011; McGowan, 1989). Although low, here we found DNA yield to be sufficient and quality to be good—increased yield could possibly be achieved by selecting only the largest feathers.

Interestingly, we found heterogeneity in sample quality dependent on location. Specifically, fecal samples from location 3 showed poor overall performance, and when it was removed from the analysis, fecal samples showed greater DNA quality than feathers. Further investigation is needed to explain this trend. For example, the abundance of PCR inhibitors could be due to subtle differences during collection, a consequence of the lower target‐to‐total DNA ratio, a diet or soil effect, or the loose consistency of kiwi scats. Idaghdour et al. (2003) also found that insect remains in feces inhibited DNA amplification. While time since the collection has not been found to have an impact on inhibition (Baumgardt et al., 2013), DNases have been linked with DNA degradation (Regnaut et al., 2006). These factors should be taken into account when working with wild kiwi, and we stress the importance of preserving DNA appropriately from the moment of collection.

Low‐template DNA samples may also exacerbate heterogeneity in locus performance. Here, five loci performed poorly for the fecal samples, which may be due to their short primer sequence and relatively long amplicons (Table S1, Appendix S1) (Opel, Chung, & McCord, 2010). Removing these five loci reduces the discriminatory power of the marker panel in all species, as indicated by the higher probability of identity values (Table 1). However, they still show higher power than necessary for individualization according to a threshold proposed by Peakall et al. (2006), although it is possible that the required discriminatory power may still not be achieved. Relatedness studies of *A. owenii* at least will likely be problematic with the reduced marker panel, in accordance with previous studies that have shown low genetic diversity within this species (Ramstad et al., 2013).

Finally, in addition to the technical challenges described above, the utility of a noninvasive genetic method is dependent on the accessibility and integrity of the noninvasive samples. For kiwi, we do not yet know how long feathers and scats can be exposed to environmental conditions before DNA quality falls below useful thresholds. Also, finding adequate numbers of samples in the wild will not be trivial. While molted feathers can be found around kiwi burrows and on hook grass (*Uncinia* spp.) (Rogan Colbourne pers. comm.), it is not clear whether they can be found in sufficient numbers and of adequate integrity. To help, a sampling device for feathers could be designed that would allow the passive collection of plucked feathers, as has been done elsewhere (Mowat & Strobeck, 2000; Patko et al., 2016), such as sticky traps placed at well‐used burrow entrances. Further, collection of feathers could be undertaken at times of year that increase discovery rates, for example, when birds are nesting or weather is amenable. Locating feces will likely be particularly challenging; however, using specifically trained detection dogs is a realistic possibility (Beebe, Howell, & Bennett, 2016; Duarte et al., 2016; Long, Donovan, Mackay, & Zielinski, 2007; Orkin, Yang, Yang, Yu, & Jiang, 2016; Rolland et al., 2006). These considerations will form the research questions of future work, along with validation of the marker panel to assess population growth and decline.

Although it comes with its own unique set of challenges, noninvasive DNA sampling, either directed or opportunistic, addresses several of the problems associated with current kiwi monitoring methods and could be particularly useful for initial data accumulation from remote areas for which less is known, as well as ongoing monitoring of managed populations. Furthermore, data retrieved from the genetic monitoring tool could supplement and/or verify data obtained from other monitoring methods such as geographical and call‐based individual identification methods already established for *Apteryx mantelli* and *A. haastii* (Corfield, Gillman, & Parsons, 2008; Dent & Molles, 2016). Our proposed protocol for collecting kiwi feces and sample collection for optimal DNA preservation is similar to those used in other surveys in New Zealand (Appendix S2 (Ramón‐Laca et al., 2014)), so presumably, its uptake by conservation officers would be relatively straightforward. Moreover, the noninvasive methodology developed here may well be transferable to other cryptic and hard‐to‐monitor bird species facing similar conservation challenges.

In summary, we have, for the first time, described a powerful and wide‐reaching, noninvasive genetic method for monitoring kiwi, with the power to discriminate between all kiwi taxa, as well as between individuals and sex. Furthermore, a DNA extraction method has been optimized for the recovery of sufficient quality DNA from noninvasive fecal and feather samples. To the best of our knowledge, this is the first study using fecal DNA in ratites, and the first study thoroughly assessing the quality and quantity of nuclear DNA from avian feces. Our methodology should therefore be valuable for use in ongoing monitoring and conservation of kiwi, and other cryptic aves.

## CONFLICT OF INTEREST

None declared.

## DATA ACCESSIBILITY

Additional Methods, Results, Discussion, and Tables S1 and S2 in Appendix S1. Collection instructions in Appendix S2. Complete marker performance results in Table S3.

## AUTHOR CONTRIBUTIONS

ARL and DJW contributed equally to this manuscript. ARL conceived the study and performed laboratory work. DJW and ARL designed the experiments, analyzed data, and wrote the manuscript. JTW and HAR provided the samples and made significant intellectual contributions to the manuscript.
