# Supplementary material for: Extraction of DNA from captive‐sourced feces and molted feathers provides a novel method for conservation management of New Zealand kiwi (Apteryx spp.)
Source: Ecol Evol. 2018 Feb 17;8(6):3119–30. doi: 10.1002/ece3.3795 (PMC5869209; doi:10.1002/ece3.3795)
Supplement: Supplementary file 1 [file ECE3-8-3119-s001.docx]

**File S1 - Additional Methods, Results and Discussion**

**Extraction of DNA from captive-sourced faeces and moulted feathers provides a novel method for conservation management of New Zealand kiwi (Apteryx spp.) by Ana Ramón-Laca, Daniel J. White, Jason T. Weir, Hugh A. Robertson**

**Methods**

*Optimisation of non-invasive sample collection and DNA extraction*

A total of 100 fresh (≤ 1-day-old) faecal samples that had been protected from rainfall were collected from three captive breeding facilities: Westshore Wildlife Reserve (Napier), Auckland Zoo (Auckland), and Kiwi Encounter (Rainbow Springs, Rotorua). Samples were taken from aviaries that were cleaned the day before to ensure samples were all less than one day old. Samples were collected by trained staff and the authors, and put in either >96 % ethanol (Fig. 2a) or in a stabilising buffer (Fig. 2b; Longmire *et al.* 1997) immediately on collection to avoid any biotic, hydrolytic, enzymatic or microbial degradation that could cause ADO. To avoid any bias at the sampling step, the order of the two collection methods was alternated. Samples then underwent two different DNA extraction approaches.

The 100 samples were split into four equal sub-groups of twenty-five. The first sub-group used one whole dropping per sample stored in >96 % ethanol and the QIAamp DNA Stool Mini Kit (Qiagen) (Fig. 2a). The second sub-group also used one whole dropping stored in >96% ethanol, but this time the PowerSoil DNA Isolation kit (Mo Bio Laboratories) was used to purify DNA (Fig. 2a). For both cases, up to 0.25 g of the outermost layer of the scats was used as starting material for DNA extraction. For the third and fourth sub-groups (Fig. 2b), a rayon swab was used to collect faecal material from the surface of the stool (see instructional video: http://youtu.be/zniEFYLSgOI) (Bosnjak *et al.* 2013; Ramón-Laca *et al.* 2014) and the head of the swab was preserved in ca 500 µl of lysis buffer (Longmire *et al.* 1997). The urine-associated, white part of the scat was avoided whenever possible, as it was found to inhibit the PCR in a small pilot study (results not shown – also see Segelbacher and Steinbruck (2001)). For the latter two sets of samples, 220 µl of the suspension and 4.2 µl of digestive enzyme (Qiagen) were used for the DNA extraction after overnight incubation at 56 °C, the difference being that the third sub-group followed a phenol-chloroform DNA extraction (Sambrook *et al.* 1989) and the fourth used an automated extraction in a QIAxtractor instrument using DX reagents (Qiagen). DNA extracted from all approaches was eluted in 70 µl of the appropriate buffer (Qiagen elution buffer was used for the phenol/chloroform batch).

*Quantification of the DNA*

Total DNA quantity of samples from the four different faecal treatments was measured using a QuantiFluor-ST Handheld fluorometer (Promega) and a Picogreen dsDNA dye kit (Quant-iT, Invitrogen, by Life Technologies). Extractions using scat scrapings preserved in ethanol (Fig 2a) were not subjected to further quantification tests or quality assessments due to low DNA yield.

DNA extracted from moulted feather samples and faecal swab samples (stored in lysis buffer) was then subjected to target DNA quantification, enabling target-to-total DNA ratio to be estimated for the faecal samples. A monomorphic microsatellite, KMS16B (Jensen *et al.* 2008) of 148 base pairs, was used to quantify the target DNA in a quantitative PCR (qPCR) approach. Ten serial dilutions from 5 to 0.002 ng µl^–1^ were used as standards for the quantification. All samples and standards were run in triplicate on a Rotor-Gene 6000 (Corbett Research) with a first holding step of 5 min at 95 °C, followed by 45 cycles of 5 s at 95 °C, 30 s at 60 °C, 30 s at 72 °C, and a final melting step from 60 to 90 °C to evaluate the specificity of the reaction. PCR mixes consisted of 5 µl of LightCycler 480 SYBR Green I mix (Roche), 0.5 µM each of KMS16B forward (CCCCCCACTAAGTCTG) and reverse (AAGTATTCTTGGTAAACAGG) primers, 0.4 µg µl^–1^ of bovine serum albumin (BSA) and 1 µl of the DNA template in a 10 µl reaction. Samples that failed to amplify were diluted up to 10-fold and re-run.

*PCR inhibition assessment*

To assess the level of inhibition in the faecal DNA extracts a similar test to the target DNA quantification experiment was conducted, except for a lower BSA concentration (0.15 µg µl^–1^) and the 2.5 ng µl^–1^ standard was included in the PCR mix as an inhibition control (1 µl per sample). Samples were run in duplicate and the 2.5 ng µl^–1^ positive control (no DNA or standard added) was run in triplicate. Inhibition occurrence was defined as the average C_q_ (quantification cycle) of the replicates for each sample minus the average C_q_ of the positive control replicates (ΔC_q_). Samples are expected to show negative results in absence of inhibitors and positive values when PCR inhibitors were co-purified in the DNA extraction. The total number of cycles (45) was used to calculate ΔCq for those samples that failed to amplify. Inhibition occurrence tests were not performed for the shed feather samples since no PCR inhibitors were assumed to be co-purified in the DNA extraction procedure.

*DNA quality assessment*

To assess the quality of DNA, DNA extracts from the moulted feathers and both swab treatments were subjected to microsatellite profiling (22 loci) and sex determination (EST-derived microsatellite locus) (Table 1) in quadruplicate. Multiplexed PCR amplifications were performed as for the panel optimisation, but with 2 µl of each DNA extract and 45 cycles in the PCR. All microsatellite fragments from non-invasive and invasive samples were scored and edited using GeneMapper v 5.0 (Applied Biosystems). DNA quality was assessed with the consensus Quality Index (QI) (Miquel *et al.* 2006) as described in Ramón-Laca *et al.* (2015). Genotyping error rates across replicates were estimated using GIMLET v 1.3.3 (Valière 2002), in which errors were classed as discrepancies of each replica to its consensus profile, or to the consensus profile of the reference feather sample when available. To validate the use of faecal and moulted feather samples for population monitoring, non-invasive genotypes were included in a principal coordinates analysis (PCoA) with genotypes generated from reference samples of high quality source (blood, tissue and plucked feathers) using birds of known origin. Any reference sample that failed at more than two loci was removed from the analysis.

**Results**

*DNA quantification of non-invasive samples*

Total DNA concentrations from extractions using the QIAamp DNA Stool Mini Kit (Qiagen) and PowerSoil DNA Isolation kit (Mo Bio Laboratories) were close to 0 ng µl^–1^ (results not shown) and thus were not analysed further. Swabbed samples extracted using the automated and phenol-chloroform approaches, yielded 12.67 (±19.46 SD) ng µl^–1^ and 5.24 (±7.84 SD) ng µl^–1^, respectively.

Target nuclear DNA concentration for the swabbed samples using the automated treatment was 9.4 (±13.2 SD) pg µl^–1^ for all three sampling locations and 6.8 (±7.2 SD) pg µl^–1^ for the two sampling locations that did not show blatant inhibition (1 and 2). Target DNA recovery was highly variable among sampling locations since target-to-total ratios for locations 1, 2 and 3 were 0.896 %, 8.102 % and 0.027 %, respectively. Shed feather samples yielded 237 (±421 SD) pg µl^–1^ of target DNA. Most phenol-chloroform extracted samples did not amplify and thus their target DNA concentration was not calculated.

*PCR inhibition assessment from faecal samples*

PCR inhibitors were found in 92 % of the samples from the phenol-chloroform treatment. ΔC_q_ was 8.2 (±5.38 SD) when only the few samples that showed amplification in the inhibition qPCR assay are considered (n=9, 36 %), and ΔC_q_ = 16.16 (±8.1 SD) when samples that failed to amplify are considered (where positive ΔCq values represent degree of inhibition). For the automated treatment, ΔC_q_ = 1.74 (±6.84 SD) with 6, 24 % of the samples showing inhibition, which was more recurrent in one of the three sampling locations (location 3). ΔC_q_ is reduced to –0.94 (±0.99 SD) if location 3 is excluded, with evidence of inhibitors in only one out of 14 samples.

*DNA quality from non-invasive samples*

Using 22 microsatellites and the sex determination marker, genotype quality (QI) for the swabbed faecal samples from the automated treatment was 0.64 (±0.36) for all locations and 0.88 (±0.13) if only locations 1 and 2 were considered. When the five microsatellite loci that had the lowest QI score were removed from analysis (KMS30, KMS14B, KMS18, KMS7R, and KMS1), QI was 0.69 (±0.4) when all 3 locations were considered and 0.93 (±0.35) for locations 1 and 2 (Fig 3). We considered a genotype to be successful when its QI was 0.75 or higher. According to this criterion, 52 % of the samples were successfully genotyped for 23 loci across all locations, and 71 % if only locations 1 and 2 were considered (Table S1). Of the samples, 68 % were successfully genotyped for 18 loci, and 92 % if only locations 1 and 2 were considered. QI for the feather samples was 0.74 (±0.23) for all 23 loci and 0.79 (±0.14) for 18 loci. Of the shed feather samples 57.7 % were successfully genotyped (QI > 0.75) for both 18 and 23 loci.

**Discussion**

Four different DNA extraction methods were tested in this study for faecal DNA purification, with only the swabbed faeces with automated extraction approach yielding DNA of sufficient quantity and quality for genotyping purposes. The remarkably low concentration of total DNA obtained from faeces preserved in ethanol and extracted using commercial kits may be due to the number of necessary pipetting steps (Barta *et al.* 2014).

As in Segelbacher (2001), we have found an obvious inhibition effect on the genotyping experiment when using the phenol-chloroform extraction protocol. Further studies would be needed to determine whether this is due to the inability of the method to remove the uric acid and other PCR inhibitors or to leftover reagents used in the extraction procedure.

Table S1 List of primers used in the multiplex and the QI results for faecal and moulted feather samples

|  |  |  |  | **Primer sequence (5’-3’)** | |  |  |  |  |
| --- | --- | --- | --- | --- | --- | --- | --- | --- | --- |
| **M** | **Locus** | **Reference** | **5’ dye** | **Forward** | **Reverse** | **[µM]** | **QIs_3_** | **QIs_2_** | **QI_f_** |
| 1 | Z37B | (Dawson *et al*. 2015) | 6-FAM | AACTGGTTGTAGGTATAGTGCAATTATG | GATTACAAAGCCAATATGGATGC | 0.15 | 0.68 | 0.98 | 0.94 |
| 2a | KMS18 | (Jensen *et al*. 2008) | NED | TGCCTTCTCTGCTTGAG | ATCCTCCAAATGCCC | 0.4 | 0.46 | 0.75 | 0.41 |
| 1 | KMS7R |  | PET | GCTTGTCCCTTTAGATTTAGCGG | TGTTTTCCCTCCTACTCAATGCTC | 0.45 | 0.48 | 0.82 | 0.71 |
| 1 | KMS14B |  | 6-FAM | GCTAACATTCACTTGGCATC | TGAATCCCTTGGATACTGAGA | 0.25 | 0.46 | 0.79 | 0.60 |
| 1 | KMS37 |  | PET | TTCCAGAGCACACACTTAG | GCATAGAACTCACATTTGC | 0.1 | 0.66 | 0.95 | 0.81 |
| 2a | KMS30 |  | NED | CTGTCAAAATCATCTTTACCAC | TTTCTCTGAGTTTCCGTCC | 0.4 | 0.31 | 0.50 | 0.40 |
| 1 | KMS1 |  | VIC | AAAGCAGCCAAGTTTTTC | TGAATGGAGTCAAGGAAG | 0.2 | 0.5 | 0.77 | 0.73 |
| 2b | Apt29 | (Shepherd & Lambert 2006) | 6-FAM | AGTAGCTACATGCGTACGTGTC | TGGCCCACCTGGAGATGTGCA | 0.15 | 0.73 | 0.96 | 0.93 |
| 2b | Apt35 |  | VIC | CAGCTTGTCTCAGGGAGCATTTGT | CTATCTCAAGCGGCATCACAAAAG | 0.4 | 0.63 | 0.86 | 0.68 |
| 2a | Apt37 |  | NED | CTGATTTGGCTTACTGCTGAC | AAGGCTGAATCCAGGCCAA | 0.15 | 0.71 | 0.96 | 0.73 |
| 2a | Apt59 |  | PET | TCTGTGCCTTGGAAGCAGTC | GGAAGCTTGGGATCACTGGG | 0.4 | 0.64 | 0.91 | 0.58 |
| 2a | Apt68 |  | 6-FAM | GGACCAGTGTGTTTATATATTCTGC | TGCAGATTCAGCCAGTAACG | 0.2 | 0.63 | 0.91 | 0.88 |
| 2b | Aptowe1 | (Ramstad *et al.* 2010) | NED | AAATCTCCTTATTAAAGAAACAGAGG | AACTCCACCGTTGTGAAAATG | 0.15 | 0.69 | 0.89 | 0.88 |
| 2b | Aptowe3 |  | PET | CCCTGGAAATTTAACATTAGGC | AATCCTCCAATTCTGACAGAGG | 0.15 | 0.63 | 0.89 | 0.60 |
| 1 | Aptowe8 |  | NED | AAGTCCATCAGCTCAGCAATC | AACAGCAAACTTAGGGACACG | 0.2 | 0.53 | 0.84 | 0.63 |
| 1 | Aptowe15 |  | PET | CCAAAAGTACTGCAGGGTTATTC | ACATGGCTTAACTGCCACTG | 0.15 | 0.66 | 0.98 | 0.88 |
| 2b | Aptowe23 |  | PET | TCTACACTGGCCAAAACAAAAC | GATCTGAACTGGCTGAAACC | 0.15 | 0.79 | 0.98 | 0.88 |
| 2b | Aptowe24 |  | VIC | TGTCACACAAAGGGCAACTC | AGGAAGCACAGGTCAGGATG | 0.15 | 0.79 | 0.96 | 0.84 |
| 2a | Aptowe28 |  | NED | GGTAAATGGGCCTCGAATAC | CAAGATGAAGACAGGTGCTTTAGAC | 0.3 | 0.63 | 0.88 | 0.80 |
| 1 | Aptowe29 |  | VIC | CCTGTCCTTTAATAATTTCTGCTC | ACTGGCCACAACAAAGCATC | 0.15 | 0.71 | 1.00 | 0.79 |
| 2b | Aptowe34 |  | 6-FAM | TTGTCCTAAACCAAAGACATGC | TTAGCATGGCTGAAAAATAGC | 0.3 | 0.59 | 0.86 | 0.68 |
| 1 | Aptowe35 |  | NED | ATCAGGTGCAGGCTGATTTC | CCAGCAGAAACAAGAACCAC | 0.3 | 0.68 | 0.95 | 0.68 |
| 1 | Aptowe39 |  | 6-FAM | AAAATAGAACCTTCCAAGCTGAAC | ATTCAGTGGGAAAAGCTTGC | 0.1 | 0.66 | 0.91 | 0.86 |

M: multiplex amplification reaction in which the primer was allocated; 5’ dye: fluorescent label added to the forward primer on the 5’ end; [µM]: final micromolar PCR primer concentration; QIs_3_: quality index per locus for the faecal swab samples from three locations studied (n=25) and DNA extracted using the automated approach (QIAxtractor); QIs_2_: quality index per locus for faecal swab samples from two locations studied (n=14) and DNA extracted using the automated approach (QIAxtractor); QI_f_: quality index per locus for the moulted feather samples (n=26)

**Table S2** Comparison of genotyping errors occurrence and methods used in peer-reviewed articles on avian species involving genotyping of faeces and moulted feathers

| **Reference** | **Species** | **Source of DNA** | **Extraction method** | **ADO**  **(%)** | **FA (%)** | **AF (%)** | **Comments** |
| --- | --- | --- | --- | --- | --- | --- | --- |
| This study | *Apteryx mantelli* | Moulted feathers | Qiagen DX reagents, automated procedure (QIAxtractor platform) | 21.9 | 0.8 | 14 | Samples eluted in 70 µl. 57.7, 68 and 92 % of samples showed QI ≥0.75 at 18 loci, respectively. |
|  |  | Faeces* |  | 20.5 | 1.3 | 28 |  |
|  |  | Faeces** |  | 16.7 | 0.8 | 6 |  |
| (Segelbacher & Steinbruck 2001) | *Tetrao urogallus* | Faeces | QIAamp DNA stool kit | - | - | - | Elution in 50 µl. |
| (Segelbacher & Storch 2002) | *Tetrao urogallus* | Moulted feathers | DNeasy tissue kit | - | - | - | Elution in 60-100 µl. 17 % of moulted feathers at 10 loci |
| (Horvath *et al*. 2005) | *Aquila adalberti* | Moulted feathers | DNeasy tissue kit | - | - | 0 | Elution in 120 µl. [DNA] = 92.19 ng µl^-1^. Negligible ADO and FA. |
| (Regnaut *et al*. 2006) | *Tetrao urogallus* | Faeces | QIAamp DNA stool kit | 21 | 3 | 10.6 | Elution in 375 µl |
| (Bayard de Volo *et al*. 2008) | *Accipiter gentilis* | Moulted feathers | Isopropanol | - | - | - | Freshly moulted. Elution in 20 µl. [DNA] = 27.3 ng µl-1 |
| (Miño & Lama 2009) | *Platalea ajaja / Jabiru mycteria* | Moulted feathers | Phenol-chloroform | 5.3 | 0 | - | Elution in 100 µl. [DNA] = 115.5 ng µl-1 for moulted remiges. |
| (Pérez *et al*. 2011) | *Tetrao urogallus* | Faeces | QIAamp DNA stool kit | 8.1 | 1.4 | - | 52.63 % of the samples amplified for at least 12 loci (of 14) |
| (Johansson *et al*. 2012) | *Lagopus lagopus* | Moulted feathers | DNeasy tissue kit | - | - | - | Elution in 50-100 µl. [DNA] = 15.8 ng µl-1. 23 % amplified at 15 loci (of 18 loci). |
| (Rösner *et al*. 2014) | *Tetrao urogallus* | Faeces | QIAamp DNA stool kit | 5.9 | 0.11 | - | 99 % success at 10 loci (only fresh samples were collected) |

ADO: allelic dropout; FA: false allele; AF: amplification failure; *for the three locations sampled; **for locations 1 and 2. ADO and FA values are described here as rates across loci

**References**

Barta J, Monroe C, Teisberg JE*, et al.* (2014) One of the key characteristics of ancient DNA, low copy number, may be a product of its extraction. *Journal of Archaeological Science* **46**, 281-289.

Bayard de Volo S, Reynolds RT, Douglas MR, Antolin MF (2008) An improved extraction method to increase DNA yield from moulted feathers. *The Condor*, **110**, 762-766.

Bosnjak J, Stevanov-Pavlovic M, Vucicevic M (2013) Feasibility of non-invasive molecular method for sexing of parrots. *Pakistan Journal of Zoology* **45**, 715-720.

Dawson DA, Brekke P, Dos Remedios N, Horsbugh GJ (2015) A marker suitable for sex-typing birds from degraded samples. *Conservation Genetics Resources*, **7**, 337–343.

Horváth MB, Martínez-Cruz B, Negro JJ, Kalmár L, Godoy JA (2005) An overlooked DNA source for non-invasive genetic analysis in birds. *Journal of Avian Biology*, **36**, 84-88.

Jensen J, Nutt KJ, Seal BS, Fernandes LB, Durrant B (2008) Isolation and characterization of microsatellite loci in the North Island brown kiwi, *Apteryx mantelli*. *Molecular Ecology Resources* **8**, 399-401.

Johansson MP, McMahon BJ, Höglund J (2012) Amplification success of multilocus genotypes from feathers found in the field compared with feathers obtained from shot birds. *Ibis*, **154**, 15-20.

Longmire JL, Maltbie M, Baker. RJ (1997) Use of “lysis buffer” in DNA isolation and its implications for museum collections. *Occasional Papers Museum of Texas Tech. University* **171**, 1-8.

Miño CI, Lama SND (2009) Molted feathers as a source of DNA for genetic studies in waterbird populations. *Waterbirds*, **32**, 322-329.

Miquel C, Bellemain E, Poillot C*, et al.* (2006) Quality indexes to assess the reliability of genotypes in studies using noninvasive sampling and multiple-tube approach. *Molecular Ecology Notes* **6**, 985-988.

Pérez T, Vázquez J, Quirós F, Domínguez A (2011) Improving non-invasive genotyping in capercaillie (*Tetrao urogallus*): redesigning sexing and microsatellite primers to increase efficiency on faeces samples. *Conservation Genetics Resources*, **3**, 483-487.

Ramón-Laca A, Gleeson D, Yockney I*, et al.* (2014) Reliable discrimination of 10 ungulate species using high resolution melting analysis of faecal DNA. *PLoS ONE* **9**, e92043.

Ramón-Laca A, Soriano L, Gleeson D, Godoy JA (2015) A simple and effective method for obtaining mammal DNA from faeces. *Wildlife Biology* **21**, 195-203.

Ramstad KM, Pfunder M, Robertson HA*, et al.* (2010) Fourteen microsatellite loci cross-amplify in all five kiwi species (Apteryx spp.) and reveal extremely low genetic variation in little spotted kiwi (*A. owenii*). *Conservation Genetics Resources*, **2**, 333-336.

Regnaut S, Lucas F, Fumagalli L (2006) DNA degradation in avian faecal samples and feasibility of non-invasive genetic studies of threatened capercaillie populations. *Conservation Genetics*, **7**, 449-453.

Rösner S, Brandl R, Segelbacher G, Lorenc T, Müller J (2014) Noninvasive genetic sampling allows estimation of capercaillie numbers and population structure in the Bohemian Forest. *European Journal of Wildlife Research*, **60**, 789-801.

Sambrook J, Fritsch EF, Maniatis T (1989) *Molecular cloning: a laboratory manual.* Cold Spring Harbor Laboratory Press, Cold Spring Harbor, NY, USA.

Segelbacher G, Steinbruck G (2001) Bird faeces for sex identification and microsattelite analysis. *Die Vogelwarte* **41**, 139-142.

Segelbacher G, Storch I (2002) Capercaillie in the Alps: genetic evidence of metapopulation structure and population decline. *Molecular Ecology*, **11**, 1669-1677.

Shepherd LD, Lambert DM (2006) Nuclear microsatellite DNA markers for New Zealand kiwi (Apteryx spp.). *Molecular Ecology Notes*, **6**, 227-229.

Valière N (2002) Gimlet: a computer program for analysing genetic individual identification data. *Molecular Ecology Notes* **2**, 377-379
