# Supplementary material for: Extraction of DNA from captive‐sourced feces and molted feathers provides a novel method for conservation management of New Zealand kiwi (Apteryx spp.)
Source: Ecol Evol. 2018 Feb 17;8(6):3119–30. doi: 10.1002/ece3.3795 (PMC5869209; doi:10.1002/ece3.3795)
Supplement: Supplementary file 2 [file ECE3-8-3119-s002.docx]

**File S2 - Kiwi DNA collection instructions**

**Extraction of DNA from captive-sourced faeces and moulted feathers provides a novel method for conservation management of New Zealand kiwi (Apteryx spp.) by Ana Ramón-Laca, Daniel J. White, Jason T. Weir, Hugh A. Robertson**

**Kiwi DNA collection instructions**

Before handling a sample remember to wear a fresh pair of gloves and/or use forceps and use a new swab/vial or envelope for each sample. Please provide a spread sheet with sample details (collection date, location, collector, comments if applicable).

Faecal DNA collection method

Only faeces of fresh appearance should be collected on non-rainy days. Collection of swab samples can be carried out by trained staff, community groups or volunteers.

Collection method

1. Remove sterile swab from its protective case/pouch

2. Submerge CLEAN swab into the 1.5-ml vial with preservation buffer* ONCE, before swabbing

3. GENTLY wipe the entire surface of the stool with the swab avoiding the white part of the stool if possible and the inside of the stool

4. Insert swab head into its 1.5-ml vial and split the handle with the aid of the lid of the vial so only the head is submerged in buffer

5. Label the lid of the 1.5-ml vial with a unique number using a permanent marker.

Discard gloves, forceps, and swab’s protective case/pouch.

A video showing how to swab the samples can be found at EcoGene®’s website: http://www.ecogene.co.nz/video.asp

*Preservation buffer is Longmire buffer (Longmire *et al.* 1997). This is a non-toxic solution (although it should not be ingested) that preserves the DNA at room temperature for long periods of time and meets requirements for air transport.

Feather DNA collection method

1. Take the feather sample from the distal end using forceps or gloves.

2. Place the feather in a new clean paper envelope.

3. Label the envelope with a unique number. Keep the envelopes in a dry place or in a bag with silica beads.

Discard gloves/forceps.
